# A Study of Disposition, Engagement, Efficacy, and Vitality of Teachers in Designing Science, Technology, Engineering, and Mathematics Education

**DOI:** 10.3389/fpsyg.2021.661631

**Published:** 2021-08-24

**Authors:** Pei-Yi Lin, Ching Sing Chai, Morris Siu-Yung Jong

**Affiliations:** ^1^Department of Education, National Kaohsiung Normal University, Kaohsiung, Taiwan; ^2^Department of Curriculum and Instruction, Centre for Learning Sciences and Technologies, The Chinese University of Hong Kong, Hong Kong, China

**Keywords:** stem education, vitality, partial least squares structural equation modeling, lesson design engagement, disposition, efficacy

## Abstract

This study proposes and tests a theoretical model of how perceptions of disposition, engagement, and efficacy of teachers for science, technology, engineering, and mathematics (STEM) e-learning can predict their sense of vitality when they designed STEM education. Upon the proposition, we developed and validated an instrument for examining the relationships between these variables. The participants were 122 secondary teachers of STEM education from Hong Kong. The instrument included four design aspects as follows: (i) disposition, (ii) lesson design engagement, (iii) efficacy for designing STEM e-learning, and (iv) vitality of teachers after attending a series of STEM professional development activities. To analyze the relationships among the variables, partial least squares structural equation modeling was employed. The disposition of teachers predicted lesson design engagement and both of these factors, in turn, predicted efficacy for designing STEM e-learning. In other words, if teachers have a high proposition toward designing learning activities, their engagement in the lesson design process may enhance their capacities in designing and implementing such activities. Also, the disposition of teachers and lesson design engagement predicted their vitality, revealing that well-suited STEM teachers should not only be able to design a STEM curriculum but also have a positive perception of STEM education.

## Introduction

With the rapid development of science and technology, the urgent need for science, technology, engineering, and mathematics (STEM) professionals has placed new demands for the educational systems worldwide (Huang and Jong, 2020). Integrative STEM education has received increasing attention in the field of education. An Organisation for Economic Co-operation and Development (OECD) report showed that STEM competencies are required not only for the nascent STEM workforce but also for solving real-world problems in daily life (OECD., 2016). Integrated STEM education has also been advocated to promote skills and competencies of 21st century among students, including inquiry skills, problem-solving, critical thinking, creativity, and innovation, as well as to develop a STEM-literate citizenry (Partnership for 21st Century Skills, 2011; English and Gainsburg, 2016). Advancing STEM competencies of students has thus become a crucial issue. This, in turn, demands that teachers develop interdisciplinary competencies in designing and facilitating STEM lessons (Lau et al., 2020).

With the proliferation of STEM education, an emerging research trend has investigated the development of K-12 teaching and teacher education in STEM education (Li et al., 2020). Researchers and educators have responded to this ongoing call to advance integrative STEM teaching and learning (English, 2016). Pedagogically, STEM education is not merely a combination of the four disciplines of STEM. Instead, current STEM lessons usually present design challenges, situations, and tasks that require students to use knowledge and skills to solve real-world problems from multidisciplinary perspectives (Feinstein and Kirchgasler, 2015; Chai et al., 2020). However, conventionally, these four disciplines have been taught in isolation. To promote changes in STEM curricula and instruction, the core content and interdisciplinary activities of STEM subjects must be connected (Henderson et al., 2011). Competency of teachers in designing an integrated STEM curriculum is crucial to develop interconnected STEM knowledge of students and to encourage students to pursue STEM-related careers (English, 2017; Timms et al., 2018). East Asian countries and regions, including Hong Kong, constantly ranked among the top 10 countries worldwide in international assessments of science and mathematics (OECD., 2016; Mullis et al., 2019). Nonetheless, in order to promote integrated understanding and improve the creativity and problem-solving ability of students, Hong Kong has launched an integrative STEM pedagogical framework (Education Bureau, 2016; Chen and Lo, 2019; Leung, 2020). The newly established curriculum framework will only be fruitful if teachers are willing to be engaged in the continuous effort needed for the design and refinement of STEM curricula (Chai et al., 2020).

To date, there are relatively few STEM studies that have been conducted in Asian contexts (Li et al., 2019). One recent study among K-12 school teachers in Hong Kong has indicated that few participants (<6%) regarded themselves as “well-prepared” for STEM education (Geng et al., 2019). Previous studies have indicated that the disposition of teachers and their lesson design practices were predictive of their efficacy in designing technology integrated lessons (Koh et al., 2015). This study explores the relationships among disposition, lesson design engagement, and lesson design efficacy of teachers, along with the vitality of teachers (in the literature review). It is based on the premise that teaching entails the design and redesign process (Kali et al., 2011; Hong et al., 2019). Current research highlights the need for teachers to be engaged in lesson design work, especially in literature that adopts the technological pedagogical content knowledge (TPACK) framework. While lesson design is generally recognized treated as demanding work (McKenney et al., 2015), the rejuvenating effects of design work among teachers have apparently been overlooked. Successful design can help teachers gain a sense that they could overcome challenges put forth by current reforms [e.g., information and communications technology (ICT) integration and interdisciplinary STEM]. In this study, the effects are epitomized as vitality. In other words, this study aims to first test a survey for its psychometric properties to measure the proposed factors, and subsequently test if the factors associated with design work could contribute to the sense of the vitality of teachers. The findings may point out to an expansion of understanding about the effects of design work and the importance of facilitating the design work of teachers during their professional development. This study could help researchers to further understand the psychological factors at play when teachers undertake the endeavor of designing STEM curricula.

## Literature Review

### Design and Design of Teachers

Scholars across different disciplines have developed a nuanced understanding of design (i.e., design knowledge and design pedagogy) and recognize that design thinking helps people explore and understand the complex nature of the design (Cross, 2001; Brown, 2008). Although the design is defined in multiple ways, it is generally a cognitive and physical process in which people respond to situations in need of solutions or situations that people desire to change. One of the accepted definitions referred design to as iterative processes in which the designers formulate understanding through initial problem representation which points to a tentative solution, and these initial representation and solution then “talk back” to the designers to stimulate further reflective understanding about the situation. Subsequently, the solution may be refined or new solutions may be formulated (Schön, 1983; Lawson, 1997; Koh et al., 2015). Several rounds of iteration may occur until an acceptable solution is chosen. Through the iterative design circles, an optimal concept is gradually formed in knowledge generation and integration activities, which is called the design thinking process. Design thinking is not only a problem-solving process; it is treated as a way of thinking that becomes a habit of mind (Cross, 2011).

Design problems are generally accepted as ill-structured problems that do not have clear problem-solving paths (Jonassen, 2000). In the context of education, designing instruction is the first necessary step to engender educational reform (Henriksen et al., 2017; Wu et al., 2019). Instructional design is widely applied as a process-centric model, for example, the analyze–design–develop–implement–evaluate model (Branch, 2009). The inception of the notion of TPACK as a theoretical framework to account for the knowledge that teachers need to create through design talk for technology integration (Koh et al., 2015; McKenney et al., 2015), process-oriented instructional design has been recast in the light of design thinking with heavy emphases on contextual considerations. Designing an interdisciplinary STEM curriculum involves multiple areas of content knowledge and multiple types of technological pedagogical knowledge. Only few teachers are well-versed in all four subject areas and teachers generally lack engineering knowledge (Al Salami et al., 2017; Chai, 2019). Interdisciplinary STEM curriculum design thus requires teachers to acquire diverse sets of knowledge and to be skillful in coordinating the multiple sources of knowledge through collaborative talk. These knowledge sources have to be synthesized and transformed into implementable classroom lessons (Chai et al., 2019). The lesson design processes are likely to be discursive, and the outcomes are uncertain. While some teachers might have a positive attitude to embrace such processes, and some teachers might resist (Le Fevre, 2014). Regardless of the disposition of teachers toward design, it seems clear that the engagement of teachers in design activities is the necessary means for them to develop the needed competencies (Lawson, 2005; Dorst, 2008).

Successful implementation of STEM curriculum depends on the attitudes of teachers toward the undertaking of the necessitated design work (i.e., disposition toward design) (Kerr, 1981; Bell, 2016; Al Salami et al., 2017). The positive attitude of teachers toward designing instruction beyond their disciplines determines their engagement in the instructional design process (i.e., lesson design engagement) (Chai and Koh, 2017) and their efficacy to integrate relevant engineering and technological concepts into science and mathematics curriculum (i.e., efficacy for designing STEM learning) (Chai et al., 2020). Chai et al. (2020) have indicated that designing technology-enhanced instruction for a single subject alone is a challenging task. Developing interdisciplinary lessons with technologies possesses a higher level of challenge. The ability of teachers to overcome the challenges in designing and integrating the subject matters contributes to their competencies. Based on the self-determination theory (Ryan and Deci, 2020), enhanced competencies contribute to the overall sense of personal well-being. This study chooses the notion of vitality, which denotes the overall motivation and well-being a person experienced (Blackwell et al., 2020), as one of the possible dependencies of the variables discussed regarding teachers in the design processes of STEM education. The aim of this study, which recruited 122 Hong Kong secondary teachers, was to investigate the interrelationships of perceptions of teachers of disposition, lesson design engagement, efficacy for designing STEM e-learning, and vitality after they had attended a series of STEM professional development activities.

### Science, Technology, Engineering, and Mathematics Education

The conventional method of learning STEM is as a collection of individual subjects, which neglects the connections between these disciplines (Bybee, 2013; Leung, 2020). STEM in education refers to both a curriculum and pedagogy. Teachers can design cross-curricular authentic problems in meaningful and relevant contexts for students to engage in such STEM learning (Hallström and Schönborn, 2019; Margot and Kettler, 2019). Essentially, STEM education should involve curriculum activities that require students to apply science and mathematics knowledge and incorporate technologies to accomplish real-world problem-solving through design. For example, students may apply STEM content knowledge and skills to construct a prototype in engineering design (Brophy et al., 2008; Fan and Yu, 2017). Therefore, STEM education focuses on preparing students with the design and design-thinking competencies required to connect scientific inquiry, mathematical thinking, technological literacy, and engineering design to solve relevant, authentic problems (Fan and Yu, 2017; Li et al., 2019). In summary, STEM learning must be relevant and authentic, and it must require students to engage in a problem-solving process. Teachers should design real-world situations that allow students to transfer knowledge and skills between STEM subjects to optimize their designs for problems. Teachers are expected to possess the capacities to design effective interdisciplinary teaching.

However, most teachers have received conventional training that focuses on teaching knowledge and skills and pays limited attention to designing meaningful and authentic learning situations (Wu et al., 2019). Design is generally classified as ill-structured problem-solving, and teachers lack experience in designing and implementing integrative STEM learning (Dorst and Cross, 2001; Chai and Koh, 2017). To address this concern, the professional development of STEM teachers needs to be investigated along with the disposition of teachers toward design and design competencies (Al Salami et al., 2017; Margot and Kettler, 2019). The literature review that follows thus includes a section on design-associated variables in relevant STEM research.

### Science, Technology, Engineering, and Mathematics Design Capacities

Science, technology, engineering, and mathematics researchers have begun to consider the design-thinking capacities of teachers (Li et al., 2019; Wu et al., 2019), but multiple challenges in designing STEM education remain to be discussed. First, teachers must develop design beliefs aimed toward student-centered, innovative instruction so that they may design appropriate curricula to map student needs, classroom activities, and instructional strategies (Yeh et al., 2015; Voogt and McKenney, 2017). Second, school curricula are most often disconnected from real-world contexts. School teachers need to prepare STEM content knowledge and skills of students, as well as their capacity to apply the knowledge and skills to authentic problems (Honey et al., 2014; Moore et al., 2014). Furthermore, many schoolteachers with separate subject specialties have limited experience in designing integrative STEM teaching or in coping with design problems spanning multiple STEM disciplines (Al Salami et al., 2017; Cavlazoglu and Stuessy, 2017). When developing and designing STEM curricula and instruction, teachers must use design-oriented approaches that encourage students to connect scientific, mathematical, engineering, and technological knowledge optimally to solve real-world problems (Bell, 2016; Falloon et al., 2020). There is an obvious need to plan for the coordination of basic and core concepts so that the interdisciplinary efforts could promote subject literacy. This is highly complex and not adequately addressed in teacher education (Chai, 2019). Therefore, researching the views of teachers on the design of STEM learning and their lesson design competencies in relevant contexts is crucial.

#### Disposition Toward Design

Disposition is defined as confidence in handling complexity and persistence in dealing with problematic situations (Halpern, 1998; Jong et al., 2020). Disposition toward design refers to the attitude of a teacher toward a design situation (Dong et al., 2015). The disposition helps teachers to remain open to the new design experience and to be tolerant of the ambitious design situation. It promotes an empathetic understanding of teachers toward the needs of students (Michlewski, 2008; Cross, 2011). Related studies have indicated that the views of teachers about their disposition toward design are significant indicators in technology integration (Koh et al., 2015; Chai and Koh, 2017). Furthermore, Chai et al. (2017) revealed that the design beliefs of teachers are significant predictors of their technological pedagogical content knowledge after they have participated in lesson design activities. STEM education entails a technology-integrated process. In this study, we expanded on previous studies about the design disposition of teachers into the STEM education context, which is more complex than integrating ICT into one subject area. Thus, we proposed and investigated the hypothesis that teachers with stronger design disposition are more inclined to design and develop STEM learning.

#### Lesson Design Engagement

Lesson design engagement refers to a design-thinking process through which designers can identify problems, empathize with the needs of users, ideate possible solutions, prototype models using promising ideas, gather feedback, and redesign (Razzouk and Shute, 2012). When teachers act as designers, they are involved in an iterative process to design, redesign, and reflect on their practices (Laurillard, 2013). The conventional role of a teacher is to deliver information and knowledge through textbooks, lessons, and activities, with less emphasis on designing a learning environment and activities that engage students in knowledge construction (Wiggins and McTighe, 2005). The key to STEM education lies in the dynamic creation of integrative knowledge and design practice. Design-thinking is essential in the lesson design processes for teachers to develop and implement integrative STEM education through conceptualizing, ideating, designing, prototyping, and evaluating outcomes, artifacts, and solutions (Li et al., 2019).

Although studies have illustrated that a well-integrated STEM education conceptual framework and professional development can help teachers to acquire the necessary expertise and promote their confidence, attitudes, knowledge, and efficacy when designing STEM instruction (Nadelson et al., 2013; Kelley and Knowles, 2016), knowledge about how lesson design engagement of teachers is related to their STEM learning and STEM curriculum design processes remains lacking. Therefore, this study examines the lesson design engagement of teachers and how this may be associated with their efficacy for designing STEM courses. Logically, engagement would enhance efficacy.

#### Efficacy for Designing STEM e-Learning

Self-efficacy is defined as the perceived capacity of a person (Bandura, 2006) and belief in their ability to successfully execute a given behavior (Beck and Ajzen, 1991). Efficacy for designing STEM e-learning refers to the belief of a person about his/her ability to work effectively through specific instructional design processes (Collier, 2002; Thibaut et al., 2018). Efficacy of teachers extends beyond their perceived personal capabilities to a more general view of their preparedness for teaching and affecting the desired student learning (Ross and Bruce, 2007; Settlage et al., 2009; Kelley et al., 2020). Efficacy of teachers for designing STEM learning can thus be considered as their self-expectations that they will be able to design tasks that require students to use STEM knowledge and skills in the context of complex situations or problem-solving processes (Honey et al., 2014). However, researchers have highlighted that relationships among mathematics (Fitzallen, 2015; Gravemeijer et al., 2017), engineering (Barrett et al., 2014; English et al., 2017), and other STEM disciplines require improvement. This points out the need to enhance the design capacity of teachers to foster the connections. Engagement in professional development activities that are targeted to design STEM curriculum activities generally improves the design capacity of teachers and hence their efficacy for designing STEM e-learning.

Although STEM research is emerging in education literature, the effectiveness of integrated STEM education for teachers and students remains underexplored (Honey et al., 2014; English, 2017). Efficacy of teachers for designing STEM e-learning is likely to influence learning outcomes and quality of students in the STEM classroom (Dilekli and Tezci, 2016; Zee and Koomen, 2016). In this study, efficacy for designing STEM e-learning refers to the efficacy of teachers to design STEM activities that are mediated by information and communication technologies.

#### Vitality

Depending on the circumstances, a person may experience both positive (e.g., lively and energetic) and negative feelings (e.g., burnout and feeling drained) (Ryan and Frederick, 1997; Farber, 2000; Skaalvik and Skaalvik, 2010; Flook et al., 2013). Vitality refers to the feeling of energy and excitement of an individual (Ryan and Frederick, 1997). Vitality is associated with the overall motivation and well-being to adapt to challenges (Miksza et al., 2019; Blackwell et al., 2020). As a person with a higher sense of disposition toward design is more open and unintimidated by the challenges, disposition toward design may be positively associated with vitality.

Teachers with high vitality are engrossed in their roles as teachers, and they have a tendency toward viewing teaching in a positive light (Intrator and Kunzman, 2007; Jong, 2019). Vitality is also related to agency and intrinsic motivation to pursue meaningful and successful teaching through enthusiasm for their work (Ryan and Frederick, 1997; Jong, 2016). Meaningful and successful teaching is premised upon strong lesson design, which is the outcome of the lesson design engagement of teachers. In other words, when teachers possess the disposition toward design, they are willing to spend time to be engaged in designing STEM learning that leads to innovative teaching (Koh et al., 2015). This could translate to successful teaching and hence contribute to the vitality of teachers. In summary, knowledge of the subject and pedagogy is insufficient for teachers; their energy, enthusiasm, and positive attitude toward designing STEM lessons and facilitating student learning are important (Blackwell et al., 2020).

Teachers with higher vitality are sympathetic to the needs of students, more dedicated to purposefully improving learning conditions, and competent in providing teaching practices to inspire and engage their students in learning. They are also resilient in their responses to problems and challenges in the classroom (Margolis and Nagel, 2006). In particular, in the field of STEM education, teachers may encounter many design problems when developing integrative STEM learning contexts (English, 2016; Li et al., 2019). It is necessary to investigate the teaching beliefs and behaviors that may be related to the energy and inspiration of teachers to overcome these problems.

### Study Aim

The aim of this study was to investigate the relationships among the disposition of teachers toward design, lesson design engagement, efficacy for designing STEM e-learning, and vitality. The disposition toward design has been introduced to assess the inclination of teachers toward a design situation. However, Lesson design engagement of teachers provides a more comprehensive view of the behaviors of teachers during the design process. The efficacy of teachers for designing STEM e-learning illustrates their expectations regarding their capability of designing a STEM e-learning context. Vitality reflects the positive feelings of teachers in terms of energy, enthusiasm, and excitement about design.

Together, this framework may provide complimentary data supporting further STEM promotions. Li et al. (2019) advocated for design and design-thinking specific to STEM teachers. Nonetheless, relatively few studies have explicitly connected these design elements with the efficacy of teachers for designing STEM courses and vitality. Thus, the research question was formulated to guide this study: How do the disposition of teachers toward design, lesson design engagement, and efficacy for designing STEM e-learning relate to vitality in the model?

## Methods

### Participants

The participants in this study were 122 secondary school teachers (77.8% male) of STEM education from Hong Kong. The mean age of the teachers was 39.8 years (SD = 9.51 years), and the mean teaching experience was 15.1 years (SD = 9.1 years). All the participants had experience in STEM teaching. They participated voluntarily in this study and completed the survey after attending a series of STEM professional development activities that engaged the teachers to design STEM activities. They were ensured that their privacy would be maintained.

### Instruments

The instrument measured four design aspects, namely, disposition toward design, lesson design engagement, efficacy for designing STEM e-learning, and teacher vitality (measured items are listed in Appendix 1). Self-reported questionnaires were used in this study. The instruments for the disposition toward design (four items, α = 0.84; e.g., “I am comfortable with the presence of uncertainty.”) and efficacy for designing STEM e-learning (five items, α = 0.91; e.g., “I can formulate in-depth discussion topics about the STEM content knowledge for students' online discussion.”) were based on the survey developed by Chai et al. (2017). The items for the efficacy for designing STEM e-learning items were adapted since the original survey was designed to investigate the efficacy of teachers for designing technological pedagogical content knowledge. Lesson design engagement items were constructed for this study to assess the STEM design effort of teachers in terms of identifying learning goals, generating teaching ideas, designing relevant STEM learning activities, and revising the design. These design-thinking activities in the lesson design processes have been identified earlier (Dick and Carey, 1996; Chai and Koh, 2017), and Koh et al. (2015) argued that teachers could create technology-integrated lessons through the design-thinking process. This study follows a design-thinking approach and constructs the lesson design engagement items. For example, one item was “I consider including new strategies that may facilitate students learning.” Vitality (four items, α = 0.80–0.89) was adopted from the study by Bostic et al. (2000) that measured the feeling of teachers of being alive and alert during the design-thinking process. An example item is “When I am engaged in lesson design, I feel alive and vital.” All items were scored on a 5-point Likert scale (i.e., 1 = strongly disagree to 5 = strongly agree). The survey was reviewed by three education professors to ensure content validity.

### Data Analysis

**Figure 1:**
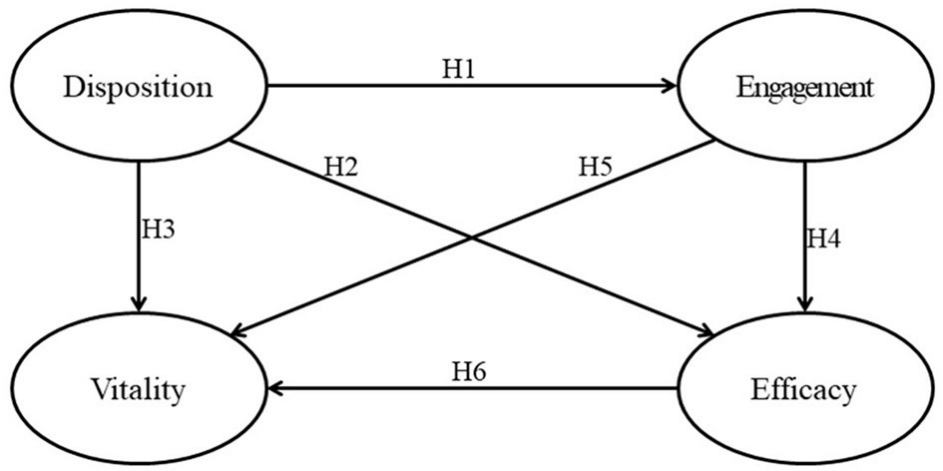
Structural equation model of the design-associated variables of teachers.

We employed partial least squares structural equation modeling (PLS-SEM) using SmartPLS 3 software (Ringle et al., 2015). PLS-SEM was used since it produces similar results to SEM, with advantages in coping with a small sample size than SEM (Hair et al., 2019). We examined the measurement and structural models following a two-step approach: validation of the measurement models and examination of the structural relations among the latent factors. The research framework contains four components, as depicted in Figure 1. The hypothesis testing was performed to assess the relationships proposed in this study as follows:

H1: Disposition toward design predicts lesson design engagement.
H2: Disposition toward design predicts efficacy for designing STEM e-learning.
H3: Disposition toward design predicts vitality.
H4: Lesson design engagement predicts efficacy for designing STEM e-learning.
H5: Lesson design engagement predicts vitality.
H6: Efficacy for designing STEM e-learning predicts vitality.

## Results

A PLS-SEM is composed of two sub-models. First, we examined the relationships between the observed data and the constructs in the measurement model, and second, we tested the hypotheses in the structural model (Hair et al., 2014).

### Measurement Model

First, the psychometric properties of the survey to establish its reliability, validity, and collinearity are assessed. The reliability of variables was examined using Cronbach's alpha and composite reliability (CR). The Cronbach's alpha values of all the constructs were from 0.78 to 0.92 (>0.7) (Table 1). All the CR values ranged from 0.86 to 0.94 (>0.7), indicating good internal consistency (Hair et al., 2014). Convergent and discriminant validities were evaluated through loadings of indicators, the average variance extracted (AVE) values, and the square root of AVEs. The loadings of the items of indicators ranged from 0.74 to 0.91 (>0.7). The AVEs of the constructs ranged from 0.60 to 0.76 (>0.5), indicating the satisfactory convergent validity (Fornell and Larcker, 1981). As shown in Table 2, the square roots of AVE for all constructs were higher than their correlation coefficients with the other constructs (Fornell–Larcker criterion), indicating that the constructs possessed good discriminant validity (Chin, 1998). Finally, variance inflation factors (VIFs) for all variables were examined to check the collinearity of the constructs. The values for VIF were from 1.41 to 3.71 (<5) (Hair et al., 2014). The outcomes of reliability, convergent validity, discriminant validity, and collinearity in the PLS-SEM analysis confirmed that the adopted and adapted items in this study were reliable and valid.

**Table 1:** Results of the measurement model.

**Latent construct**	**Item**	**Indicator loading**	***T*-value**	**Cronbach's Alpha**	**CR**	**AVE**	**VIF**
Disposition toward design	1	0.85	23.65	04	0.90	0.76	1.82
	2	0.89	41.79				2.19
	3	0.86	26.91				2.03
Lesson design engagement	1	0.74	14.13	8	0.86	0.60	1.41
	2	0.78	21.20				1.44
	3	0.81	17.34				1.76
	5	0.77	12.07				1.67
Efficacy for designing STEM e-learning	1	0.87	34.11	2	0.94	0.76	2.77
	2	0.85	24.72				3.12
	3	0.90	36.47				3.71
	4	0.91	44.86				3.67
	5	0.82	20.52				2.08
Vitality	1	0.88	38.51	9	0.92	0.75	2.50
	2	0.90	45.88				3.03
	3	0.83	13.26				2.10
	4	0.84	22.90				2.12

**Table 2:** Discriminant validity test results of the measurement model.

	**1**	**2**	**3**	**4**
1. Disposition toward design	0.87			
2. Lesson design engagement	0.44	0.77		
3. Efficacy for designing STEM e-learning	0.56	0.53	0.87	
4. Vitality	0.55	0.67	0.53	0.87

*Diagonal elements are the square roots of the average variance extracted*.

### Structural Model

**Figure 2:**
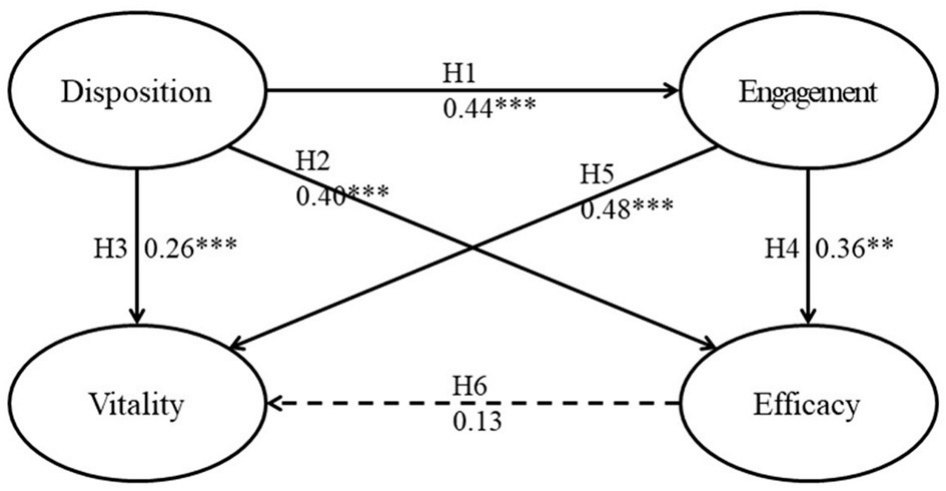
Structural model of the design-associated variables of teachers. ^**^p < 0.01, ^***^p < 0.001.

The structural model was assessed by examining the significant level of path coefficients in the model. As shown in Table 3 and Figure 2, five significant predictive relations were observed in the model with path coefficients (*β*) ranging from 0.26 to 0.48. Disposition of teachers toward design positively predicted their perceived lesson design engagement (*β* = 0.44, *p* < 0.001), efficacy for designing STEM e-learning (*β* = 0.40, *p* < 0.001), and vitality (*β* = 0.26, *p* < 0.01), and lesson design engagement of teachers positively predicted their perceptions of efficacy for designing STEM e-learning (*β* = 0.36, *p* < 0.001) and vitality (*β* = 0.48, *p* < 0.001). However, the efficacy of teachers for designing STEM e-learning did not predict vitality (*β* = 0.13, *p* > 0.05). These results showed that, when the teachers possess a higher disposition toward designing STEM learning and lesson design engagement, they may exhibit a strong sense of efficacy for designing STEM e-learning and possess higher vitality.

**Table 3:** Path estimated of the structural model.

**Path estimates (hypotheses)**	**Path coefficient**	**Mean**	**Standard deviation**	**T statistics**	***p*-value**	**Hypotheses supported?**
H1: Disposition -> Engagement	0.44	0.45	0.07	6.10	<.001	Yes
H2: Disposition -> Efficacy	0.40	0.40	0.08	5.34	<.001	Yes
H3: Disposition -> Vitality	0.26	0.26	0.09	3.04	<.001	Yes
H4: Engagement -> Efficacy	0.36	0.36	0.07	4.78	0.003	Yes
H5: Engagement -> Vitality	0.48	0.49	0.08	5.95	<.001	Yes
H6: Efficacy -> Vitality	0.13	0.13	0.08	1.59	0.11	No

## Discussions and Conclusion

Since STEM education is interdisciplinary, the design of STEM curriculum involves a high degree of complexity (Chai, 2019). Teachers need to integrate the different STEM disciplines to design meaningful teaching and learning topics and activities. However, in practice, most STEM educators lack experience in designing integrative STEM curricula, which may result in teachers having low efficacy for designing STEM e-learning and vitality. Thus, designing integrative STEM curricula is a major challenge in STEM education. In this study, a new instrument was designed to assess these design-associated variables. This study observes the key to create good STEM education depending on the design of teachers. The disposition of teachers toward design was defined as their propensity to deal with the inevitable uncertainties and ambiguities involved when they develop new pedagogies, understand the needs of students, generate teaching ideas, and design activities for STEM education. The disposition of teachers toward design facilitates iterative lesson design engagement necessary to create and refine the STEM curriculum continuously. Following (Chai and Koh, 2017) articulation, we defined the lesson design engagement of teachers as their commitment to creating new strategies and objectives and testing the ideas in relation to the learning processes of students. The iterative engagements improve the STEM lessons designed and promote the efficacy of teachers for designing STEM e-learning, which refers to their beliefs in their capabilities to design and implement effective STEM teaching strategies to bring about the desired learning outcomes. Finally, we defined vitality as the energetic feelings and excitement of teachers when designing STEM learning activities.

The results of PLS-SEM indicated a reliable measurement model with satisfactory convergent and discriminant validities. In other words, this study has validated four interrelated psychological factors that could be used to study the design-based work of teachers for integrated STEM education. Regarding the structural model, several positive associations were identified between the design beliefs, design behavior, perceived efficacy for designing STEM e-learning, and vitality of teachers in the design context as hypothesized. In particular, the disposition of teachers toward design predicted lesson design engagement, and both disposition toward design and lesson design engagement positively predicted efficacy for designing STEM e-learning and vitality. Since significant relationships were observed between the disposition of teachers toward design, lesson design engagement, and efficacy for designing STEM e-learning in this study, we considered that the teachers with a greater inclination to and involvement in the design-thinking process might possess higher expectations for effective STEM design outcomes. In general, the more design tendencies the teacher possessed, and the more engagement they reported, the higher efficacy they perceived, and the more feelings of being energized by the design activities they expressed. This is in line with previous studies showing that the design inclination of a person is likely to be positively associated with their belief in self-efficacy (Jong et al., 2020), involvement in design practice (Koh et al., 2015), and the optimistic outlook to embrace the uncertainties and ambiguities of design situation (Dong et al., 2015; Royalty et al., 2015).

The model depicts a positively associated web of factors that could help to address the need for STEM curriculum design (Hallström and Schönborn, 2019). It also implied that teacher educators might need to pay attention to understand the disposition of teachers toward design and highlight the ill-structured nature of design challenges (Margot and Kettler, 2019). This could address the expectations of teachers of what to expect when they participate in STEM curriculum design activities that could be riddled with uncertainties and ambiguities. In addition, teachers also need to understand the iterative design-thinking engagement needed over an extended period of time (Dorst and Cross, 2001). The findings indicate that the teachers with a stronger disposition toward design are more adept in being engaged in iterative design-thinking processes (Koh et al., 2015). In contrast, the model also implied that teacher developers have to provide adequate support for design thinking during the iterative processes of STEM lesson design so that the activities could foster the efficacy of teachers for designing STEM e-learning (Chai and Koh, 2017). This would likely lead to a sense of vitality, which is a positive and desirable outcome of the complex interdisciplinary design effort.

Teacher efficacy for designing STEM e-learning denotes the beliefs of teachers about their ability to design and has reciprocal relations in goal-directed STEM activities (Lent and Brown, 2006). Studies have also reported that the engagement of teachers in design activities influences their self-efficacy beliefs (Salanova et al., 2011; Simbula et al., 2011; Chai et al., 2020). In this study, the teachers were engaged in professional development. The experience of engagement could generate opportunities for a sense of mastery in designing integrative STEM activities, which is integral to developing self-efficacy beliefs (Bandura, 1997). In this view, the engagement of teachers at design work as a form of professional development may predict their perceived capability of performing design work.

Bell (2016) indicated that teachers who are energized might be capable of designing a well-integrated STEM learning context that could foster the motivation of students to learn STEM, development of problem-solving skills, and pursuit of a related degree and career. In other words, high vitality could initiate cycles of positive growth toward STEM design and teaching expertise. However, the self-efficacy of teachers may be reduced due to burnout, and promoting a healthy classroom environment relies on their high self-efficacy (Flook et al., 2013). Thus, it is important to sustain the efficacy of teachers for designing STEM e-learning and vitality for STEM education. As depicted in Figure 2, the sustenance hinges upon lesson design engagement with the disposition toward design as the predictor. Support to foster continuous lesson design engagement and disposition toward lesson design is thus important. School leaders have to offer the structure for teachers to engage in design-thinking, while teacher educators may have to play the role of supporting teachers' design thinking (Chai, 2019; Chai et al., 2020). As interdisciplinary STEM education is a complex endeavor, it should also be noted that the long-term efforts are likely to be needed.

Chai et al. (2017), Dong et al. (2015), and Li et al. (2019) have emphasized the importance of design beliefs. This study explores how the design traits of individuals impact lesson design engagement, efficacy for designing STEM e-learning, and vitality. This study observes that disposition toward design and design-thinking competencies might play a significant role in the design attitudes of teachers and involvement in designing STEM learning. Specifically, the strong disposition of teachers toward design indicates that they feel comfortable with the ambiguous design problems and that they may respond with design thinking to overcome these problems (Chai et al., 2017). The significance of the effect of disposition toward design on lesson design competencies indicated that when teachers feel capable of managing design problems, their design competencies also improved, enabling them to effectively deal with the demands of design situations (Koh et al., 2015). Teachers who possessed a stronger disposition toward design and capacity for designing STEM learning may feel excited as STEM designers (Kali et al., 2015). This finding suggests that, when confronting a new design situation that teachers have not experienced before, those with high tolerance toward ambiguity may be more engaged in regulating their design thinking to deal with the design tasks. Teachers with a high disposition toward design could thus be a good choice for school leaders when they need teachers to innovate teaching and learning. This study also implies that engaging in STEM education is essentially a design-intensive process. If educational systems or peer communities of teachers could provide professional development to facilitate the engagement in the lesson design processes, teachers could feel supported in designing STEM learning, which could subsequently increase their enthusiasm for developing and implementing STEM learning (Intrator and Kunzman, 2007; Ross and Bruce, 2007; Meijer et al., 2009).

This study has some limitations. First, the sample size was relatively small. Future studies may enlarge the research sample size. Second, the survey was a self-reported assessment. We attempted to assess the design thinking of teachers, but the design is a dynamic process that depends on the context. A valid structural model with long-term instructional intervention could be considered in future studies. Third, the aim of this study was to focus on validating the proposed conceptual model and the corresponding hypotheses. Background variables, such as gender, age, years of teaching experience, were gathered in the data collection process. These data could be further analyzed using the multigroup analysis or between-group analysis in PLS-SEM, as a means of testing predefined data groups to determine if there are significant differences in group-specific parameter estimates. Fourth, this study was conducted in the context of secondary STEM education; nevertheless, we believe that the developed model and the related work presented in this study are applicable to other educational contexts, such as learning and teaching of other subjects or interdisciplinary subjects in K-12 education. Despite these limitations, the findings of this study contribute to the literature by identifying the psychological and pedagogical determinant factors for designing STEM learning.

## Data Availability Statement

The datasets presented in this article are not readily available because requests need to be vetted by the research ethic committee. Requests to access the datasets should be directed to cschai@cuhk.edu.hk.

## Ethics Statement

The studies involving human participants were reviewed and approved by The Chinese University of Hong Kong. The patients/participants provided their written informed consent to participate in this study.

## Author Contributions

All authors listed have made a substantial, direct and intellectual contribution to the work, and have approved the submitted version of the manuscript.

## Conflict of Interest

The authors declare that the research was conducted in the absence of any commercial or financial relationships that could be construed as a potential conflict of interest.

## Publisher's Note

All claims expressed in this article are solely those of the authors and do not necessarily represent those of their affiliated organizations, or those of the publisher, the editors and the reviewers. Any product that may be evaluated in this article, or claim that may be made by its manufacturer, is not guaranteed or endorsed by the publisher.

## Appendix

**Table A1:** The four latent variables and their assessment items.

**1. Disposition toward design**
I am comfortable with the presence of uncertainty.
I am comfortable to explore conflicting ideas.
I am comfortable to deviate from established practices.
**2. Lesson design engagement**
I consider including new strategies that may facilitate students learning>.
I write down clearly the lesson objectives to be achieved.
I source for relevant information and materials to make the lesson interesting.
I conduct the lesson as planned to test out the feasibility of the lesson (*item deleted*).
I revise the lesson objectives and strategies when needed.
**3. Efficacy for designing STEM e-learning**
I can formulate in-depth discussion topics about the STEM content knowledge for students' online discussion.
I can help students to construct and share different representations of the STEM knowledge using appropriate ICT tools.
I can design online inquiry activities to guide students to make sense of the STEM knowledge with appropriate ICT tools.
I can create new activities that use a range of web-based tools to facilitate students' knowledge building for the STEM project.
I can generate new ideas about how to use technology in a pedagogically appropriate way to teach the subject matter.
**4. Vitality**
When I am engaged in lesson design, I feel alive and vital.
When I am thinking about my lesson design, I have energy and spirit.
When I am designing lesson, I nearly always feel alert and awake.
When I generate new lesson ideas, I feel energized.

*One item “I conduct the lesson as planned to test out the feasibility of the lesson.” was deleted, because the factor loading was under 0.7*.

## References

[B1] Al Salami M. K. Makela C. J. de Miranda M. A. (2017). Assessing changes in teachers' attitudes toward interdisciplinary STEM teaching. Int. J. Technol. Design Educ. 27, 63–88. 10.1007/s10798-015-9341-0

[B2] Bandura A. (1997). Self-eYcacy: The Exercise of Control. New York, NY: W. H. Freeman & Co.

[B3] Bandura A. (2006). Guide for constructing self-efficacy scales. Selfefficacy Beliefs Adolesc. 5, 307–337.

[B4] Barrett B. S. Moran A. L. Woods J. E. (2014). Meteorology meets engineering: an interdisciplinary STEM module for middle and early secondary school students. Int. J. STEM Educ. 1:6. 10.1186/2196-7822-1-6

[B5] Beck L. Ajzen I. (1991). Predicting dishonest actions using the theory of planned behavior. J. Res. Pers. 25, 285–301. 10.1016/0092-6566(91)90021-H

[B6] Bell D. (2016). The reality of STEM education, design and technology teachers' perceptions: a phenomenographic study. Int. J. Technol. Design Educ. 26, 61–79. 10.1007/s10798-015-9300-9

[B7] Blackwell J. Miksza P. Evans P. McPherson G. E. (2020). Student vitality, teacher engagement, and rapport in studio music instruction. Front. Psychol. 11:1007. 10.3389/fpsyg.2020.01007 32508726 PMC7253673

[B8] Bostic T. J. Rubio D. M. Hood M. (2000). A validation of the subjective vitality scale using structural equation modeling. Soc. Indic. Res. 52, 313–324. 10.1023/A:1007136110218

[B9] Branch R. M. (2009). Instructional Design: The ADDIE Approach. Boston: Springer.

[B10] Brophy S. Klein S. Portsmore M. Rogers C. (2008). Advancing engineering education in P-12 classrooms. J. Eng. Educ. 97, 369–387. 10.1002/j.2168-9830.2008.tb00985.x

[B11] Brown T. (2008). Design thinking. Harv. Bus. Rev. 86, 84–92. 18605031

[B12] Bybee R. W. (2013). The Case for STEM Education: Challenges and Opportunities. Arlington, VA: NSTA Press.

[B13] Cavlazoglu B. Stuessy C. (2017). Changes in science teachers' conceptions and connections of STEM concepts and earthquake engineering. J. Educ. Res. 110, 239–254.

[B14] Chai C. S. (2019). Teacher professional development for science, technology, engineering and mathematics (STEM) education: a review from the perspectives of technological pedagogical content (TPACK). Asia Pacific Educ. Res. 28, 5–13. 10.1007/s40299-018-0400-7

[B15] Chai C. S. Jong M. S. Y. Yin H. B. Chem M. Zhou W. (2019). Validating and modelling teachers' technological pedagogical content knowledge for integrative science, technology, engineering and mathematics education. Educ. Technol. Soc. 22, 61–73

[B16] Chai C. S. Koh J. H. L. (2017). Changing teachers' TPACK and design beliefs through the Scaffolded TPACK Lesson Design Model (STLDM). Learn. Res. Pract. 3, 114–129. 10.1080/23735082.2017.1360506

[B17] Chai C. S. Rahmawati Y. Jong M. S. Y. (2020). Indonesian science, mathematics, and engineering preservice teachers' experiences in STEM-TPACK design-based learning. Sustainability 12:9050. 10.3390/su12219050

[B18] Chai C. S. Tan L. Deng F. Koh J. H. L. (2017). Examining pre-service teachers' design capacities for web-based 21st century new culture of learning. Aust. J. Educ. Technol. 33, 1–20. 10.14742/ajet.3013

[B19] Chen C. W. J. Lo K. M. J. (2019). From teacher-designer to student-researcher: a study of attitude change regarding creativity in STEAM education by using makey makey as a platform for human-centred design instrument. J. STEM Educ. Res. 2, 75–91. 10.1007/s41979-018-0010-6

[B20] Chin W. W. (1998). “The partial least squares approach to structural equation modeling,” in Modern Methods for Business Research, eds G. A. Marcoulides (New York, NY: Psychology Press), 295–336.

[B21] Collier M. D. (2002). Changing the face of teaching: preparing educators for diverse settings. Teacher Educ. Q. 29, 49–59.

[B22] Cross N. (2001). Designerly ways of knowing: design discipline versus design science. Design Issues 17, 49–55. 10.1162/074793601750357196

[B23] Cross N. (2011). Design Thinking. New York, NY: Berg.

[B24] Dick W. Carey L. (1996). The Systematic Design of Instruction. New York, NY: Addison-Wesley Longman.

[B25] Dilekli Y. Tezci E. (2016). The relationship among teachers' classroom practices for teaching thinking skills, teachers' self-efficacy towards teaching thinking skills and teachers' teaching styles. Think. Skills Creat. 21, 144–151. 10.1016/j.tsc.2016.06.001

[B26] Dong Y. Chai C. S. Sang G.-Y. Koh H. L. Tsai C.-C. (2015). Exploring the profiles and interplays of pre-service and inservice teachers' technological pedagogical content knowledge (TPACK) in China. Educ. Technol. Soc. 18, 158–169.

[B27] Dorst K. (2008). Design research: a revolution-waiting-to-happen. Design Stud. 29, 4–11. 10.1016/j.destud.2007.12.001

[B28] Dorst K. Cross N. (2001). Creativity in the design process: co-evolution of problem–solution. Design Stud. 22, 425–437. 10.1016/S0142-694X(01)00009-6

[B29] Education Bureau (2016). Promotion of STEM Education: Unleashing Potential in Innovation. Hong Kong: Curriculum Development Bureau.

[B30] English L. D. (2016). STEM education K-12: perspectives on integration. Int. J. STEM Educ. 3:3. 10.1186/s40594-016-0036-1

[B31] English L. D. (2017). Advancing elementary and middle school STEM education. Int. J. Sci. Math. Educ. 15, 5–24. 10.1007/s10763-017-9802-x

[B32] English L. D. Gainsburg J. (2016). “Problem solving in a 21st-century mathematics curriculum,” in Handbook of International Research in Mathematics Education, eds L. D. English and D. Kirshner, 3rd Edn (New York, NY: Taylor & Francis), 313–335.

[B33] English L. D. King D. Smeed J. (2017). Advancing integrated STEM learning through engineering design: sixth-grade students' design and construction of earthquake resistant buildings. J. Educ. Res. 110, 255–271. 10.1080/00220671.2016.1264053

[B34] Falloon G. Hatzigianni M. Bower M. Forbes A. Stevenson M. (2020). Understanding K-12 STEM education: a framework for developing STEM literacy. J. Sci. Educ. Technol. 29, 369–385. 10.1007/s10956-020-09823-x32836879

[B35] Fan S. C. Yu K. C. (2017). How an integrative STEM curriculum can benefit students in engineering design practices. Int. J. Technol. Design Educ. 27, 107–129. 10.1007/s10798-015-9328-x

[B36] Farber B. A. (2000). Treatment strategies for different types of teacher burnout. J. Clin. Psychol. 56, 675–689. 10.1002/(SICI)1097-4679(200005)56:5<675::AID-JCLP8>3.0.CO;2-D 10852153

[B37] Feinstein N. W. Kirchgasler K. L. (2015). Sustainability in science education*?* How the Next Generation Science Standards approach sustainability, and why it matters. Sci. Educ. 99, 121–144. 10.1002/sce.21137

[B38] Fitzallen N. (2015). “STEM education: what does mathematics have to offer?,” in Mathematics Education in the Margins. Proceedings of the 38th annual conference of the Mathematics Education Research Group of Australasia, Sunshine Coast, June 28-July 2, ed M. Marshman (Sydney: MERGA), 237–244.

[B39] Flook L. Goldberg S. B. Pinger L. Bonus K. Davidson R. J. (2013). Mindfulness for teachers: a pilot study to assess effects on stress, burnout, and teaching efficacy. Mind Brain Educ. 7, 182–195. 10.1111/mbe.12026 24324528 PMC3855679

[B40] Fornell C. Larcker D. F. (1981). Evaluating structural equation models with unobservable variables and measurement error. J. Mark. Res. 18, 39–50. 10.1177/002224378101800104

[B41] Geng J. Jong M. S. Y. Chai C. S. (2019). Hong Kong teachers' self-efficacy and concerns about STEM education. Asia Pacific Educ. Res. 28, 35–45. 10.1007/s40299-018-0414-1

[B42] Gravemeijer K. Stephan M. Julie C. Lin F. L. Ohtani M. (2017). What mathematics education may prepare students for the society of the future? Int. J. Sci. Math. Educ. 15, 105–123. 10.1007/s10763-017-9814-6

[B43] Hair J. F. Risher J. J. Sarstedt M. Ringle C. M. (2019). When to use and how to report the results of PLS-SEM. Eur. Bus. Rev. 31, 2–24. 10.1108/EBR-11-2018-0203

[B44] Hair J. F. Sarstedt M. Hopkins L. Kuppelwieser V. G. (2014). Partial least squares structural equation modeling (PLS-SEM): an emerging tool in business research. Eur. Bus. Rev. 26, 106–121. 10.1108/EBR-10-2013-0128

[B45] Hallström J. Schönborn K. J. (2019). Models and modelling for authentic STEM education: reinforcing the argument. Int. J. STEM Educ. 6:22. 10.1186/s40594-019-0178-z

[B46] Halpern D. F. (1998). Teaching critical thinking for transfer across domains: disposition, skills, structure training, and metacognitive monitoring. Am. Psychol. 53, 449–455. 10.1037/0003-066X.53.4.449 9572008

[B47] Henderson C. Beach A. Finkelstein N. (2011). Facilitating change in undergraduate STEM instructional practices: an analytic review of the literature. J. Res. Sci. Teach. 48, 952–984. 10.1002/tea.20439

[B48] Henriksen D. Richardson C. Mehta R. (2017). Design thinking: a creative approach to educational problems of practice. Think. Skills Creat. 26, 140–153. 10.1016/j.tsc.2017.10.001

[B49] Honey M. Pearson G. Schweingruber H. (2014). STEM Integration in K-12 Education: Status, Prospects and an Agenda for Research. Washington DC: National Academies Press.

[B50] Hong H. Y. Lin P. Y. Chai C. S. Hung G. T. Zhang Y. (2019). Fostering design-oriented collective reflection among preservice teachers through principle-based knowledge building activities. Comput. Educ. 130, 105–120. 10.1016/j.compedu.2018.12.001

[B51] Huang B. Jong M. S. Y. (2020). “Developing a generic rubric for evaluating students' works in STEM education,” in Proceedings of the 6th International Symposium on Educational Technology (ISET 2020) (Online, Bangkok, Thailand), 210–213.

[B52] Intrator S. M. Kunzman R. (2007). The person in the profession: Renewing teacher vitality through professional development. The Education Forum. 71, 16–32.

[B53] Jonassen D. H. (2000). Toward a design theory of problem solving. Educ. Technol. Res. Dev. 48, 63–85. 10.1007/BF02300500

[B54] Jong M. S. Y. (2016). Teachers' concerns about adopting constructivist online game-based learning in formal curriculum teaching. Br. J. Educ. Technol. 47, 601–617. 10.1111/bjet.12247

[B55] Jong M. S. Y. (2019). Sustaining the adoption of gamified outdoor social enquiry learning in high schools through addressing teachers' emerging concerns: a three-year study. Br. J. Educ. Technol. 50, 1275–1293. 10.1111/bjet.12767

[B56] Jong M. S. Y. Geng J. Chai C. S. Lin P. Y. (2020). Development and predictive validity of the computational thinking disposition questionnaire. Sustainability 12:4459. 10.3390/su12114459

[B57] Kali Y. Goodyear P. Markauskaite L. (2011). Researching design practices and design cognition: contexts, experiences and pedagogical knowledge-in-pieces. Learn. Media Technol. 36, 129–149. 10.1080/17439884.2011.553621

[B58] Kali Y. McKenney S. Sagy O. (2015). Teachers as designers of technology enhanced learning. Instr. Sci. 43, 173–179. 10.1007/s11251-014-9343-4

[B59] Kelley T. R. Knowles J. G. (2016). A conceptual framework for integrated STEM education. Int. J. STEM Educ. 3, 1–11.

[B60] Kelley T. R. Knowles J. G. Holland J. D. Han J. (2020). Increasing high school teachers self-efficacy for integrated STEM instruction through a collaborative community of practice. Int. J. STEM Educ. 7, 1–13. 10.1186/s40594-020-00211-w

[B61] Kerr S. T. (1981). How teachers design their materials: implications for instructional design. Instr. Sci. 10, 363–378. 10.1007/BF00162734

[B62] Koh J. H. L. Chai C. S. Hong H. Y. Tsai C. C. (2015). A survey to examine teachers' perceptions of design dispositions, lesson design practices, and their relationships with technological pedagogical content knowledge (TPACK). Asia Pacific J. Teacher Educ. 43, 378–391. 10.1080/1359866X.2014.941280

[B63] Lau W. W. F. Jong M. S. Y. Cheng G. K. S. Chu S. K. W. (2020). “Teachers' concerns about STEM education in Hong Kong,” in Proceedings of EdMedia + Innovate Learning [Online, The Netherlands: Association for the Advancement of Computing in Education (AACE)], 344–347. Available online at: https://www.learntechlib.org/primary/p/217319/ (accessed January 08, 2021).

[B64] Laurillard D. (2013). Teaching as a Design Science: Building Pedagogical Patterns for Learning and Technology. London, UK: Routledge.

[B65] Lawson B. (1997). How Designers Think: The Design Process Demystified. Oxford: Architectural Press.

[B66] Lawson B. (2005). How Designers Think: The Design Process Demystified, 4th edn. Oxford: Architectural Press.

[B67] Le Fevre D. M. (2014). Barriers to implementing pedagogical change: the role of teachers' perceptions of risk. Teach. Teach. Educ. 38, 56–64. 10.1016/j.tate.2013.11.007

[B68] Lent R. W. Brown S. D. (2006). Integrating person and situation perspectives on work satisfaction: a social-cognitive view. J. Vocat. Behav. 69, 236–247. 10.1016/j.jvb.2006.02.006

[B69] Leung A. (2020). Boundary crossing pedagogy in STEM education. Int. J. STEM Educ. 7:15. 10.1186/s40594-020-00212-9

[B70] Li Y. Schoenfeld A. H. Graesser A. C. Benson L. C. English L. D. Duschl R. A. (2019). Design and design thinking in STEM education. J. STEM Educ. Res. 2, 93–104. 10.1007/s41979-019-00020-z PMC7234448 32838129

[B71] Li Y. Wang K. Xiao Y. Froyd J. E. (2020). Research and trends in STEM education: a systematic review of journal publications. Int. J. STEM Educ. 7, 1–16. 10.1186/s40594-020-00207-6

[B72] Margolis J. Nagel L. (2006). Education reform and the role of administrators in mediating teacher stress. Teach. Educ. Q. 33, 143–159.

[B73] Margot K. C. Kettler T. (2019). Teachers' perception of STEM integration and education: a systematic literature review. Int. J. STEM Educ. 6, 1–16. 10.1186/s40594-018-0151-2

[B74] McKenney S. Kali Y. Markauskaite L. Voogt J. (2015). Teacher design knowledge for technology enhanced learning: an ecological framework for investigating assets and needs. Instr. Sci. 43, 181–202. 10.1007/s11251-014-9337-2

[B75] Meijer P. C. Korthagen F. A. Vasalos A. (2009). Supporting presence in teacher education: the connection between the personal and professional aspects of teaching. Teach. Teach. Educ. 25, 297–308. 10.1016/j.tate.2008.09.013

[B76] Michlewski K. (2008). Uncovering design attitude: inside the culture of designers. Organ. Stud. 29, 373–392.

[B77] Miksza P. Evans P. McPherson G. E. (2019). Wellness among university-level music students: a study of the predictors of subjective vitality. Musicae Sci. 25:102986491986055. 10.1177/1029864919860554

[B78] Moore T. J. Stohlmann M. S. Wang H. H. Tank K. M. Glancy A. Roehrig G. H. (2014). “Implementation and integration of engineering in K-12 STEM education,” in Engineering in Precollege Settings: Research Into Practice, eds J. Strobel, S. Purzer, and M. Cardella (Rotterdam: Sense Publishers).

[B79] Mullis I. V. S. Martin M. O. Foy P. Kelly D. L. Fishbein B. (2019). HIGHLIGHTS: TIMSS 2019 International Results in Mathematics and Science. Available online at: https://timss2019.org/reports/highlights/ (accessed January 08, 2021).

[B80] Nadelson L. S. Callahan J. Pyke P. Hay A. Dance M. Pfiester J. (2013). Teacher STEM perception and preparation: Inquiry-based STEM professional development for elementary teachers. J. Educ. Res. 106, 157–168.

[B81] OECD. (2016). PISA 2015 Technical Report. PISA. Paris: OECD Publishing.

[B82] Partnership for 21st Century Skills (2011). Framework for 21st Century Learning 2-Page. Available online at: http://www.p21.org/our-work/p21-framework (accessed January 06, 2021).

[B83] Razzouk R. Shute V. (2012). What is design thinking and why is it important? Rev. Educ. Res. 82, 330–348. 10.3102/0034654312457429

[B84] Ringle C. M. Wende S. Becker J.-M. (2015). SmartPLS3. Bönningstedt: SmartPLS. Available online at: www.smartpls.com (accessed August 06, 2021).

[B85] Ross J. Bruce C. (2007). Professional development effects on teacher efficacy: results of randomized field trial. J. Educ. Res. 101, 50–60. 10.3200/JOER.101.1.50-60

[B86] Royalty A. Ladenheim K. Roth B. (2015). “Assessing the development of design thinking: from training to organizational application,” in Design Thinking Research, eds H. Plattner, C. Meinel, and L. Leifer (Heidelberg: Springer), 73–86.

[B87] Ryan R. M. Deci E. L. (2020). Intrinsic and extrinsic motivation from a self-determination theory perspective: definitions, theory, practices, and future directions. Contemp. Educ. Psychol. 61:101860. 10.1016/j.cedpsych.2020.101860

[B88] Ryan R. M. Frederick C. (1997). On energy, personality, and health: subjective vitality as a dynamic reflection of wellbeing. J. Pers. 65, 529–565. 10.1111/j.1467-6494.1997.tb00326.x 9327588

[B89] Salanova M. Llorens S. Schaufeli W. B. (2011). “Yes, I can, I feel good, and I just do it!” On gain cycles and spirals of efficacy beliefs, affect, and engagement. Appl. Psychol. 60, 255–285. 10.1111/j.1464-0597.2010.00435.x

[B90] Schön D. A. (1983). The Reflective Practitioner: How Professionals Think in Action. New York, NY: Basic Books.

[B91] Settlage J. Southerland S. A. Smith L. K. Ceglie R. (2009). Constructing a doubt-free teaching self: self-efficacy, teacher identity, and science instruction within diverse settings. J. Res. Sci. Teach. 46, 102–125. 10.1002/tea.20268

[B92] Simbula S. Guglielmi D. Schaufeli W. B. (2011). A three-wave study of job resources, self-efficacy, and work engagement among Italian schoolteachers. Eur. J. Work Org. Psychol. 20, 285–304. 10.1080/13594320903513916

[B93] Skaalvik E. M. Skaalvik S. (2010). Teacher self-efficacy and teacher burnout: a study of relations. Teach. Teach. Educ. 26, 1059–1069. 10.1016/j.tate.2009.11.00124765710

[B94] Thibaut L. Knipprath H. Dehaene W. Depaepe F. (2018). The influence of teachers' attitudes and school context on instructional practices in integrated STEM education. Teach. Teach. Educ. 71, 190–205. 10.1016/j.tate.2017.12.014

[B95] Timms M. Moyle K. Weldon P. Mitchell P. (2018). Challenges in STEM Learning in Australian Schools. Policy Insights 7. Victoria: Australian Council for Educational Research. Available online at: https://research.acer.edu.au/policyinsights/7/ (accessed January 16, 2021).

[B96] Voogt J. McKenney S. (2017). TPACK in teacher education: are we preparing teachers to use technology for early literacy? Technol. Pedagogy Educ. 26, 69–83. 10.1080/1475939X.2016.1174730

[B97] Wiggins G. P. McTighe J. (2005). Understanding by Design. Alexandria, Egypt: Association for Supervision and Curriculum Development.

[B98] Wu B. Hu Y. Wang M. (2019). Scaffolding design thinking in online STEM preservice teacher training. Br. J. Educ. Technol. 50, 2271–2287. 10.1111/bjet.12873

[B99] Yeh Y. F. Lin T. C. Hsu Y. S. Wu H. K. Hwang F. K. (2015). Science teachers' proficiency levels and patterns of TPACK in a practical context. J. Sci. Educ. Technol. 24, 78–90. 10.1007/s10956-014-9523-7

[B100] Zee M. Koomen H. M. (2016). Teacher self-efficacy and its effects on classroom processes, student academic adjustment, and teacher well-being: a synthesis of 40 years of research. Rev. Educ. Res. 86, 981–1015. 10.3102/0034654315626801

